# Interactions Between Tsetse Endosymbionts and *Glossina pallidipes* Salivary Gland Hypertrophy Virus in *Glossina* Hosts

**DOI:** 10.3389/fmicb.2021.653880

**Published:** 2021-05-28

**Authors:** Güler Demirbas-Uzel, Antonios A. Augustinos, Vangelis Doudoumis, Andrew G. Parker, George Tsiamis, Kostas Bourtzis, Adly M. M. Abd-Alla

**Affiliations:** ^1^Insect Pest Control Laboratory, Joint FAO/IAEA Programme of Nuclear Techniques in Food and Agriculture, Vienna, Austria; ^2^Laboratory of Systems Microbiology and Applied Genomics, Department of Environmental Engineering, University of Patras, Agrinio, Greece

**Keywords:** *Hytrosaviridae*, tsetse microbiota, virus transmission, *Wigglesworthia*, *Sodalis*, *Wolbachia*

## Abstract

Tsetse flies are the sole cyclic vector for trypanosomosis, the causative agent for human African trypanosomosis or sleeping sickness and African animal trypanosomosis or nagana. Tsetse population control is the most efficient strategy for animal trypanosomosis control. Among all tsetse control methods, the Sterile Insect Technique (SIT) is one of the most powerful control tactics to suppress or eradicate tsetse flies. However, one of the challenges for the implementation of SIT is the mass production of target species. Tsetse flies have a highly regulated and defined microbial fauna composed of three bacterial symbionts (*Wigglesworthia, Sodalis* and *Wolbachia*) and a pathogenic *Glossina pallidipes* Salivary Gland Hypertrophy Virus (GpSGHV) which causes reproduction alterations such as testicular degeneration and ovarian abnormalities with reduced fertility and fecundity. Interactions between symbionts and GpSGHV might affect the performance of the insect host. In the present study, we assessed the possible impact of GpSGHV on the prevalence of tsetse endosymbionts under laboratory conditions to decipher the bidirectional interactions on six *Glossina* laboratory species. The results indicate that tsetse symbiont densities increased over time in tsetse colonies with no clear impact of the GpSGHV infection on symbionts density. However, a positive correlation between the GpSGHV and *Sodalis* density was observed in *Glossina fuscipes* species. In contrast, a negative correlation between the GpSGHV density and symbionts density was observed in the other taxa. It is worth noting that the lowest *Wigglesworthia* density was observed in *G. pallidipes*, the species which suffers most from GpSGHV infection. In conclusion, the interactions between GpSGHV infection and tsetse symbiont infections seems complicated and affected by the host and the infection density of the GpSGHV and tsetse symbionts.

## Introduction

Tsetse flies (Diptera: Glossinidae) are medically and agriculturally important vectors of trypanosomes, the causative agents of human African trypanosomosis or sleeping sickness and African animal trypanosomosis or nagana in some 37 countries throughout Sub-Saharan Africa (Leak, 1998; Simarro et al., 2003). The presence of tsetse and trypanosomes are considered as one of the most important roots of hunger and poverty in humans, hindering the adoption of more productive livestock (Dyck et al., 2021; Feldmann et al., 2021). No effective vaccines are available for disease control and existing nagana drugs are a significant financial burden for livestock owners. It is hard to manage the disease in humans by treating with trypanocidal drugs due to drug toxicity and the extended treatment times required, and resistance by the animal trypanosomes to the available drugs is common (Aksoy and Rio, 2005). Conversely, disease vector eradication using vector control methods is cheap and effective for the sustainable management of the diseases (Leak, 1998; Simarro et al., 2003). Among available vector control methods (Jordan, 1974; Thompson et al., 1991; Green, 1994), the sterile insect technique (SIT) is considered very powerful for the sustainable management of the disease as part of area-wide integrated pest management (Vreysen et al., 2000; Hendrichs et al., 2007). SIT is based on the mass production, radiation-based sterilization and release of sterile insects over a target area to suppress or locally eliminate a target insect pest population (Dyck et al., 2021).

A prerequisite for large-scale SIT applications is the sustainable production and maintenance of large numbers of high-quality insects of the target species to use during the implementation of SIT (Vreysen et al., 2000). However, this may sometimes be quite challenging due to the presence of pathogens and parasites such as the salivary gland hypertrophy virus (GpSGHV) (Abd-Alla et al., 2021).

Despite the extensive research on the ecology, physiology, genetics, and reproductive biology of tsetse flies that has been carried out over the past years, there is little knowledge about the interactions between the GpSGHV, the tsetse symbionts and the host in *Glossina* species other than *G. pallidipes* (Boucias et al., 2013). Therefore, it is crucial to investigate this tripartite interaction in order to improve the production and the quality of the mass reared insects that are to be used for SIT applications.

Tsetse flies are known to harbor a unique bacterial community mainly consisting of the obligate *Wigglesworthia glossinidia*, the commensal *Sodalis glossinidius*, and the widespread symbiont *Wolbachia pipientis* (hereafter *Wolbachia*) (Aksoy, 2000; Aksoy et al., 2008; Wang et al., 2013; Doudoumis et al., 2017). The prevalence of these symbionts in natural populations of different tsetse species may vary and some individuals may only carry the primary symbiont *Wigglesworthia glossinidia* (hereafter *Wigglesworthia*) (Lindh and Lehane, 2011). *Wigglesworthia*, a member of the *Entrobacteriaceae* family, is an obligate mutualistic bacterium, primarily residing intracellularly within the bacteriome organ in the anterior midgut of tsetse (Aksoy, 1995) and extracellularly in the mother‘s milk gland secretion (Attardo et al., 2008; Belda et al., 2010). Its symbiotic role is crucial in immunity as well as in the metabolic provisioning of vitamins and other nutrients to the hematophagous insect host which are either lacking or are contained in low amounts in vertebrate blood (International Glossina Genome Initiative, 2014; Attardo et al., 2019). Elimination of *Wigglesworthia* from tsetse results in significantly reduced host fecundity due to the loss of these vitamins while its presence during the immature stage is vital for immune system development in adults (Weiss et al., 2011).

The facultative commensal *Sodalis glossinidius* (hereafter *Sodalis*; family *Enterobacteriaceae*) is found both intracellularly and extracellularly in many different tissues in tsetse, e.g., hemolymph, midgut, and milk glands (Attardo et al., 2008; Belda et al., 2010). The *Sodalis* genome sequence shows reduced coding capacity with a large number of fragmented predicted protein-coding sequences and pseudogenes that are non-functional. Therefore, *Sodalis* cannot survive outside its host and has transitioned from a free-living form to a mutualistic life cycle (Toh et al., 2006). Even though its elimination has been reported to reduce host longevity and there are reports which suggest that *Sodalis* may render tsetse flies susceptible to trypanosome infection (Welburn and Maudlin, 1992; Dale and Welburn, 2001; Dale and Moran, 2006; Geiger et al., 2007; Farikou et al., 2010), the specific role of this symbiont is still not clear in tsetse flies.

Populations of tsetse species may also be infected with another symbiont, *Wolbachia*, which is an obligatory intracellular maternally transmitted alphaproteobacterium (Cheng et al., 2000; Doudoumis et al., 2012). *Wolbachia* has been shown to affect many aspects of the biology of its hosts, including host reproduction, development, immunity and behavior (Saridaki and Bourtzis, 2010; Schneider et al., 2011). The presence of this symbiont in the tsetse fly *G. morsitans* has been associated with the induction of cytoplasmic incompatibility, a form of reproductive abnormality which is expressed as embryonic lethality when an infected male mates with an uninfected female or a female infected with a different strain of the symbiont (Alam et al., 2011; Doudoumis et al., 2013). Due to their importance, tsetse fly-symbiont interactions are being harnessed toward the development of novel approaches for vector and disease control (Aksoy, 2000; Aksoy et al., 2008; Abd-Alla et al., 2013; Doudoumis et al., 2013).

Tsetse flies can also be infected by the salivary gland hypertrophy virus that was first found in *G. pallidipes* and hence named GpSGHV. The GpSGHV belongs to the *Hytrosaviridae* family, which are rod-shaped viruses containing a single circular double-stranded DNA genome (Abd-Alla et al., 2009b; Kariithi et al., 2019). GpSGHV causes both asymptomatic (latent/persistent) and symptomatic infections (Lietze et al., 2010; Kariithi et al., 2013). Infection with GpSGHV results in salivary gland hypertrophy (SGH) syndrome which is characterized by the swelling of the salivary glands as well as abnormalities in the reproductive organs and associated reduction on the fecundity and fertility of insect hosts (Jaenson, 1978; Abd-Alla et al., 2010a, 2009a).

In natural populations, GpSGHV is transmitted from mother to progeny, either transovum or *via* infected milk glands (Jura et al., 1989; Sang et al., 1996, 1998). In laboratory conditions, GpSGHV infection occurs horizontally via membrane feeding and vertically from mother to offspring (Abd-Alla et al., 2010b). Interestingly, *G. pallidipes* is highly susceptible to intra-hemocoelic GpSGHV injection. Although this infection route results in high viral titers (≥ 10^9^ viral genome copies), it triggers the onset of overt SGH only in the F1 offspring, not in the injected adults. Notably, although GpSGHV has been detected in almost all *Glossina* species that have so far been PCR-screened, the occurrence of SGH symptoms is a very rare event. For example, PCR detection of GpSGHV in *G. pallidipes* shows widespread asymptomatic virus infection (up to 100%), with only 5–10% of the infected individuals developing SGH symptoms. However, symptomatic infections in *G. pallidipes* tsetse fly mass production factories may result to dramatic reductions in fecundity leading to colony collapse (Abd-Alla et al., 2011). It is unclear why some infected flies show symptomatic infection whereas others remain asymptomatically infected. Although a positive correlation was found between symptomatic SGH and increased virus copy number (which indicates an accumulation effect of the virus related to the SGH symptoms), other unknown factors related to the fly’s genetic background and interaction with its microbiota (symbionts) cannot be excluded (Moran et al., 2008; Abd-Alla et al., 2009a; Ferrari and Vavre, 2011; Boucias et al., 2013; Eleftherianos et al., 2013). Therefore, it is important to analyze the interaction between the virus and the symbiotic bacteria in different tsetse species.

Based on the variable responses to the GpSGHV infection in different tsetse species and in the lack of molecular explanation, the role of tsetse microbiota in modulating GpSGHV infection was not excluded. Analyzing the interactions between GpSGHV and tsetse symbionts might reveal the mechanism behind the different responses to GpSGHV infection in different tsetse species. To date, the interactions between GpSGHV and the bacterial symbionts in the *Glossina* species remain an open question. Similar to the maternally-transmitted *Wigglesworthia*, *Sodalis* and *Wolbachia* (Rio et al., 2012), GpSGHV is trans-ovarially transmitted, and the absence of these symbionts can change the outcome of the virus infection (Boucias et al., 2013).

In the current study, we performed a series of assays to examine the levels of GpSGHV infection and its interaction with the three endosymbionts *Wigglesworthia*, *Sodalis*, and *Wolbachia* in laboratory colonies of six tsetse species. The disentangling of the potential diverse interactions among the different microbial species occurring in the six tsetse species studied is discussed in the context of developing an effective and robust mass production system of high-quality sterile tsetse flies for implementing SIT programs.

## Materials and Methods

### Tsetse Samples

All the GpSGHV injection related laboratory experiments described in this study were conducted on tsetse fly colonies maintained at the Insect Pest Control Laboratory, Joint FAO/IAEA Centre of Nuclear Techniques in Food and Agriculture, Seibersdorf, Austria. The laboratory colonies of the six *Glossina* taxa used in the injection experiments originated from Uganda (*G. pallidipes*), Kenya (*G. brevipalpis*), Zimbabwe (*G. m. morsitans*), Tanzania (*G. m. centralis*), Central African Republic (*G. f. fuscipes*), and Burkina Faso (*G. p. gambiensis*).

Experimental flies were held in standard round holding cages (20 cm diameter × 5 cm height) at a density of 45–80 flies (male: female ratio of 1:3) based on the species size and maintained at 23 ± 1°C and 75–80% RH under the insectaria conditions. Experimental flies were fed on heated, defibrinated bovine blood (10–15 min; three times weekly) using the *in vitro* membrane feeding technique (Feldmann, 1994; Gooding et al., 1997).

### *In vivo* Virus Replication in Intra-Hemocoelic Injected Adults

A pair of intact salivary glands dissected from 10-day old male *G. pallidipes* exhibiting overt salivary gland hypertrophy symptoms were used to prepare the virus inoculum as previously described (Boucias et al., 2013) with slight modifications including aseptic salivary gland dissections and use of non-filtered virus inoculum.

Virus injection was conducted in the different tsetse species as previously described by Demirbas-Uzel et al. (2018b). In brief, teneral flies were immobilized in the chiller (2–6°C; 5 min) and injected (intra-hemocoelic) with either 2 μl sterile phosphate buffered saline (PBS) or GpSGHV virus suspension. For each species, 160 flies (1:3 male:female) were injected and placed in standard holding cages at a density of 45–80 flies per cage. The experiments were conducted in 2–3 replicates for each species. After the injections, eight flies (six females and two males) were randomly selected and sampled from both virus and PBS injected fly groups at 0-, 1-, 5-, and 9-days post injection (dpi), and subsequently frozen at −20°C until further analysis. Virus density variations were measured separately for both females and males at different dpi.

### Extraction of Total DNA and PCR Amplifications

The total DNA of each sample was extracted from whole bodies of individual flies using the DNeasy tissue kit (QIAGEN Inc., Valencia, CA) following the manufacturer’s instructions. The DNA extracted was eluted in 200 μl of the elution buffer. Then, 30 μl of the extracted genomic DNA from individual samples were pooled (six females in one pool and two males in another pool), and the DNA concentrations in the pooled DNA were determined using a spectrophotometer (Synergy H1 Multi-Mode Reader, BioTek, Instruments, Inc., United States). The pooled DNAs were diluted to obtain equal final DNA concentrations (4 ng/μl) and 5 μl of the diluted DNA was analyzed by qPCR on a CFX96 real time PCR detection system (Bio-Rad, Hercules, CA) as described previously (Abd-Alla et al., 2009a). The tsetse housekeeping β*-tubulin* gene was used to normalize the qPCR reactions (Boucias et al., 2013) to give the virus density. The primers and the qPCR conditions are given in Supplementary Table 1.

The aliquots of the DNA extracted at the time-points outlined in section “*In vivo* Virus Replication in Intra-Hemocoelic Injected Adults” were used to quantify the densities of bacterial symbionts normalized to the housekeeping β*-tubulin* gene as previously described (Boucias et al., 2013). *Sodalis, Wigglesworthia* and *Wolbachia* densities were quantified in both males and females and at different dpi by qPCR using primers that target *fliC* (Weiss et al., 2012), *thiC* (Yamada et al., 2007) and *Wolbachia* 16R rRNA genes, respectively (Demirbas-Uzel et al., 2018a). The primers and the PCR and qPCR conditions are given in Supplementary Table 1.

### Statistical Analysis

The density of *Wigglesworthia*, *Sodalis* and *Wolbachia* in GpSGHV infected flies was evaluated in both PBS and GpSGHV injected flies at 0, 1, 5, and 9 dpi and was normalized against the average of the zero-time density by dividing the density of each reading at the targeted point by the average of the density at zero-time. Subsequently, the density of each targeted point of the flies injected with GpSGHV was normalized against the average of each time point of the PBS-injected flies. The densities of *Wigglesworthia*, *Sodalis* and *Wolbachia* in GpSGHV injected flies normalized against the PBS-injected densities were then used in the statistical analysis. The GpSGHV density data previously reported by Demirbas-Uzel et al. (2018b) was reanalyzed using the above method and compared with the tsetse symbiont density. Data were checked for normality and transformed where necessary using the Box-Cox transform (x^λ^ − 1)/λ. The significance of the overall differences of the virus and symbiont densities obtained from the various treatments were assessed by ANOVA (Sokal and Rohlf, 1995), after which the actual patterns of differences between the means were determined by Tukey’s honestly significant difference (HSD) test. The *P*-values were calculated from the data with the significance threshold selected as 0.05.

The analyses were executed in R v4.0.2 (R Core Team, 2020) using RStudio v1.3.1056 (Baier and Neuwirth, 2007; RStudio Team, 2016) with packages ggplot2 v3.3.2.1 (Wickham, 2016), lattice v0.20-41 (Sarkar, 2008) and MASS v7.3-51.6 (Venables and Ripley, 2002). All regression analyses were conducted using the linear model (lm) for different times and different doses and coefficient factors (slope), *t* and *P*-values are presented in Supplementary Table 2. Overall similarities in *Wigglesworthia, Sodalis*, *Wolbachia* and GpSGHV density between tsetse species, time post injection and sex were shown using the multidimensional scaling (MDS) analysis, and the multidimensional plots as implemented in PRIMER version 7+ (Anderson, 2001; Clarke and Gorley, 2016). Permutational multivariate analysis of variance (PERMANOVA) was applied to Bray-Curtis similarity matrices to compute similarities between countries, tsetse species and infection type groups using PRIMER version 7+.

## Results

### Impact of GpSGHV Infection on the Three Bacterial Symbionts in Different Tsetse Species

**FIGURE 1:**
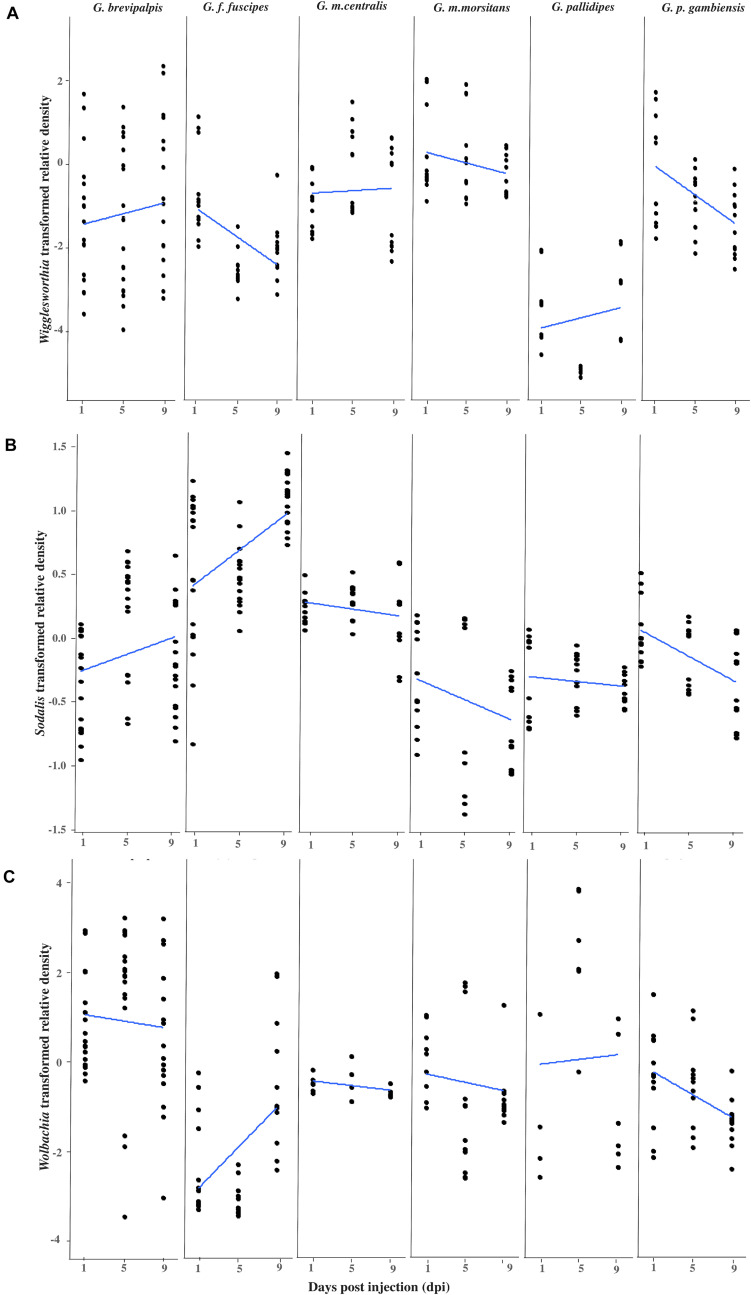
Impact of GpSGHV infection on the density of tsetse symbionts. Transformed *Wigglesworthia* **(A)**, *Sodalis* **(B)**, and *Wolbachia* **(C)** densities for six taxa of tsetse flies injected with GpSGHV normalized against control flies. Total DNA was extracted from six females and two males at 1, 5, and 9 days post injection and used as template for qPCR. Data were normalized to the housekeeping β*-tubulin* gene, against time zero and against the control injected flies at each time point and transformed based on the Box-Cox lambda value. The linear regression line was calculated using the lm method in R.

After the intra-hemocoelic injections of virus suspension into the teneral adults, all six tsetse taxa were found to be susceptible to GpSGHV infection under laboratory conditions and the virus relative density increased over time in most taxa (Supplementary Figure 1). After confirming the virus infection, the relative qPCR quantification of *Wigglesworthia*, *Sodalis* and *Wolbachia* densities over a 9-day experimental period revealed various densities of these symbionts across the six *Glossina* taxa (Figure 1).

#### Impact of GpSGHV on *Wigglesworthia* Density

The density of *Wigglesworthia* (*Wig*D) in virus injected flies relative to PBS injected flies varied significantly between tsetse taxa (Taxon: F = 42.855, *df* = 5.206, *P* < 0.001), with time over 9 days post injection (Time: F = 5.3002, *df* = 1.206, *P* = 0.022), as well as the interactions between sex and taxon (*F* = 7.014, *df* = 5.206, *P* < 0.001) and time and taxon (*F* = 3.318, *df* = 5.206, *P* = 0.007; Figure 1A and Supplementary Table 2). In general, *Wig*D in GpSGHV injected adults was not affected by the fly sex (*F* = 0.023, *df* = 1.206, *P* = 0.881; Supplementary Table 2) but a significant difference was observed between males and females in *G. brevipalpis* (*F* = 9.280 *df* = 1.52 *P* = 0.003) and *G. m. centralis* (*F* = 14.781 *df* = 1.32 *P* = 0.001; Supplementary Figure 1A and Supplementary Table 3). GpSGHV significantly reduced *Wig*D over time post injection in *G. f. fuscipes* (Coefficient = −0.165, *P* = 0.001) and *G. p. gambiensis* (Coefficient = −0.170, *P* = 0.002). In contrast, no significant change was observed in *Wig*D over time after injection in the other taxa (Supplementary Table 4). In general, *Wig*D was lower in *G. pallidipes* than the other tsetse species (Figure 1). The greatest reduction effect by GpSGHV on *Wig*D was seen in *G. f. fuscipes* (*P* = 0.001; Figure 1A and Supplementary Table 4).

#### Impact of GpSGHV on *Sodalis* Infection Density

Overall, GpSGHV effects on *Sodalis* density (*Sod*D) were significantly different between the six tsetse taxa (*F* = 70.593, *df* = 5.228, *P* < 0.001). Although no significant difference was observed between males and females (*P* = 0.129) or with time (*P* = 0.350), strong interactions between sex and time (*F* = 5.819, *df* = 1.228, *P* = 0.017), sex and taxon (*F* = 4.417, *df* = 5.228, *P* < 0.001) and time and taxon (*F* = 8.778, *df* = 5.228, *P* < 0.001) were observed (Supplementary Table 2).

*Sodalis* density was evaluated within each of the six taxa, but only in *G. f. fuscipes* did it increase significantly with time over the 9 days post injection (Coefficient = 0.071, *P* < 0.001) and reduced significantly in *G. p. gambiensis* (Coefficient = −0.050, *P* < 0.001; Figure 1B and Supplementary Table 4).

No significant difference was observed between females and males in respect to *Sod*D over all tsetse taxa (*F* = 2.321, *df* = 1.228, *P* = 0.129; Supplementary Table 2) but a significant difference was observed between males and females within *G. m. centralis* (*F* = 32.208, *df* = 1.32, *P* < < 0.001) and *G. p. gambiensis* (*F* = 46.091, *df* = 1.24, *P* < < 0.001; Supplementary Table 3).

#### Impact of GpSGHV on *Wolbachia* Infection Density

The difference in *Wolbachia* density (*Wol*D) was significant only between different taxa (*F* = 20.868, *df* = 5.189, *P* < < 0.001), while there was no significant difference between males and females (*P* = 0.238), between the flies from different time points post infection (*P* = 0.796) or any interaction term (Figure 1C and Supplementary Table 2). Over time, in the presence of GpSGHV infection, a significant increase in *Wol*D was observed in *G. f. fuscipes* (Coefficient = 0.224, *P* = 0.004) and a significant reduction was observed in *G. p. gambiensis* (Coefficient = −0.125, *P* = 0.009) over 9 days post injection compared to control flies. No significant changes were observed in the other taxa (Supplementary Table 4). No significant difference was observed between males and females when all species were analyzed together, Supplementary Table 3.

### Interaction Between GpSGHV and Tsetse Symbionts

**FIGURE 2:**
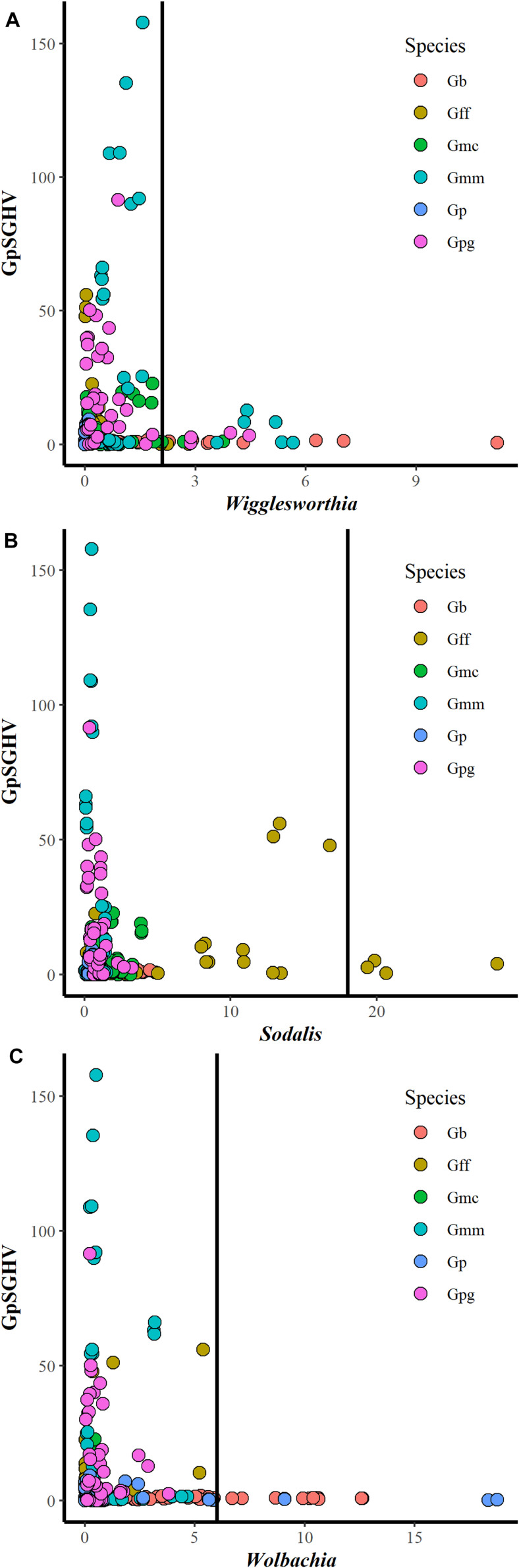
Interaction between GpSGHV relative density and the relative density of *Wigglesworthia* **(A)**, *Sodalis* **(B),** and *Wolbachia* **(C)** in six taxa of tsetse flies. Tsetse adults were injected with GpSGHV and the density of GpSGHV and tsetse symbionts were analyzed by qPCR and the data were normalized against control flies and zero time of injection. The vertical bar indicates the tsetse symbiont relative density to limit the relative density of GpSGHV to 20.

**FIGURE 3:**
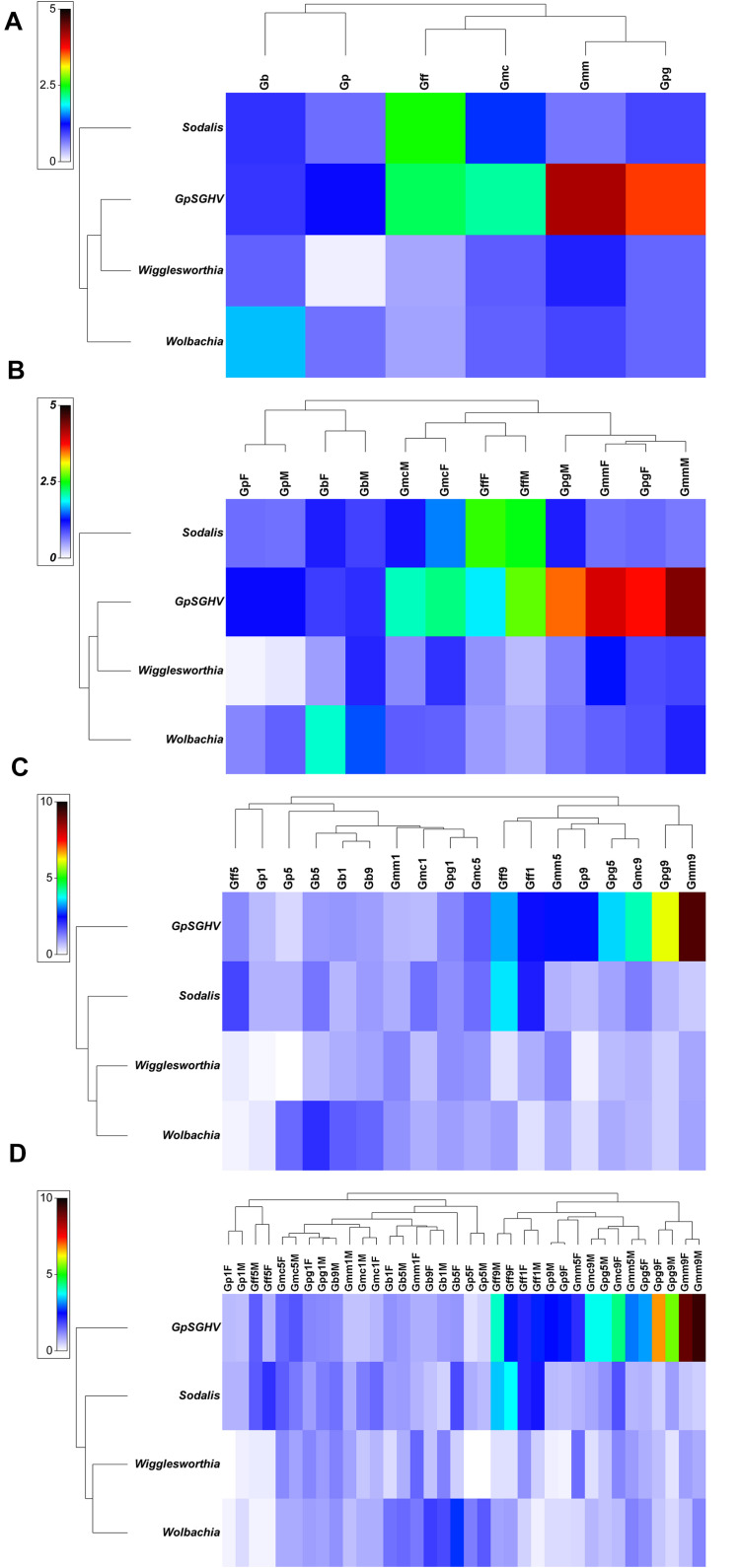
Relative density of GpSGHV, *Wigglesworthia*, *Sodalis* and *Wolbachia* in six taxa of tsetse flies. Tsetse adults were injected with GpSGHV and the density of GpSGHV and tsetse symbionts were analyzed by qPCR and the data were normalized against control flies and zero time of injection. Data were transformed to square root and averaged based on tsetse species **(A)**, tsetse species and sex **(B)**, tsetse species and time post GpSGHV injection **(C)**, and tsetse species, time post GpSGHV injection and sex **(D)**. Gb, *Glossina brevipalpis*; Gff, *G. f. fuscipes*; Gmc, *G. m. centralis*; Gmm, *G. m. morsitans*; Gp, *G. pallidipes*; Gpg, *G. p. gambiensis*. Time post GpSGHV injection: 1, 5, and 9 dpi. F: female, M: male.

Comparing the GpSGHV relative density with the relative density of tsetse symbionts indicated that this multi-partite interaction seems to be complicated and varied from one tsetse species to another. Plotting the GpSGHV relative density against tsetse symbiont densities indicate a negative correlation between the GpSGHV and both *Wigglesworthia* and *Wolbachia*. The same trend was observed in *Sodalis* in most of the tested taxa except *G. fuscipes* which show that a high density of GpSGHV can be observed with high density of *Sodalis* (Figure 2). Analyzing the similarity between GpSGHV and tsetse symbiont densities indicated that (i) the lowest density of *Wigglesworthia* was opserved in *G. pallidipes*, (ii) the highest increase if GpSGHV density was observed in *G. m. morsitans* and *G. p. gambiensis*, (iii) the highest density of *Sodalis* was found in *G. f. fuscipes*, and (iv) the highest density of *Wolbachia* was observed in *G. brevipalpis* (Figure 3A). These previous statement gets more complicated when sex and time post GpSGHV injection or both of them are also taken into consideration. The lowest *Wigglesworthia* density in *G. pallidipes* was associated with a higher density of GpSGHV than the density of both *Sodalis* and *Wolbachia*. The GpSGHV density and *Sodalis* density did not show a visible difference between males and females, however, the density of *Wigglesworthia* and *Wolbachia* was slightly higher in males than females (Figure 3B). The highest density of *Sodalis* observed in *G. f. fuscipes* was not affected by the sex of the flies but was affected by the time post GpSGHV injection. The dinsity of the GpSGHV and tsetse symbionts were affected by the time post GpSGHV injection, at 1 dpi the density of GpSGHV and *Sodalis* were similar and higher than the density of *Wigglesworthia* and *Wolbachia*, however at 5 dpi the density of *Sodalis* remained unchanged but the GpSGHV density decreased and the *Wolbachia* density increased. This situation was changed at 9 dpi where the GpSGHV density increased and *Wolbachia* density decreased (Figure 3C). In G. morsitans, the highest GpSGHV density was associated with a low density of *Sodalis*, which was lower than *Wolbachia* density and *Wigglesworthia*. This situation was affected by the sex of the flies where the GpSGHV density was visibly higher in males than females with slightly higher density of *Wolbachia* than *Wigglesworthia*, as opposed to the females with lower GpSGHV density associated with higher density of *Wigglesworthia* than *Wolbachia.* The highest GpSGHV density was mainly observed at 5 and 9 dpi with low density of the tsetse symbiont. It is worth noting that tsetse symbiont density was not affected by the time post injection (Figure 3C). The highest density of *Wolbachia* observed in *G. brevipalpis* was mainly observed in the females and was not affected by the time post GpSGHV injection (Figures 3B,D).

**FIGURE 4:**
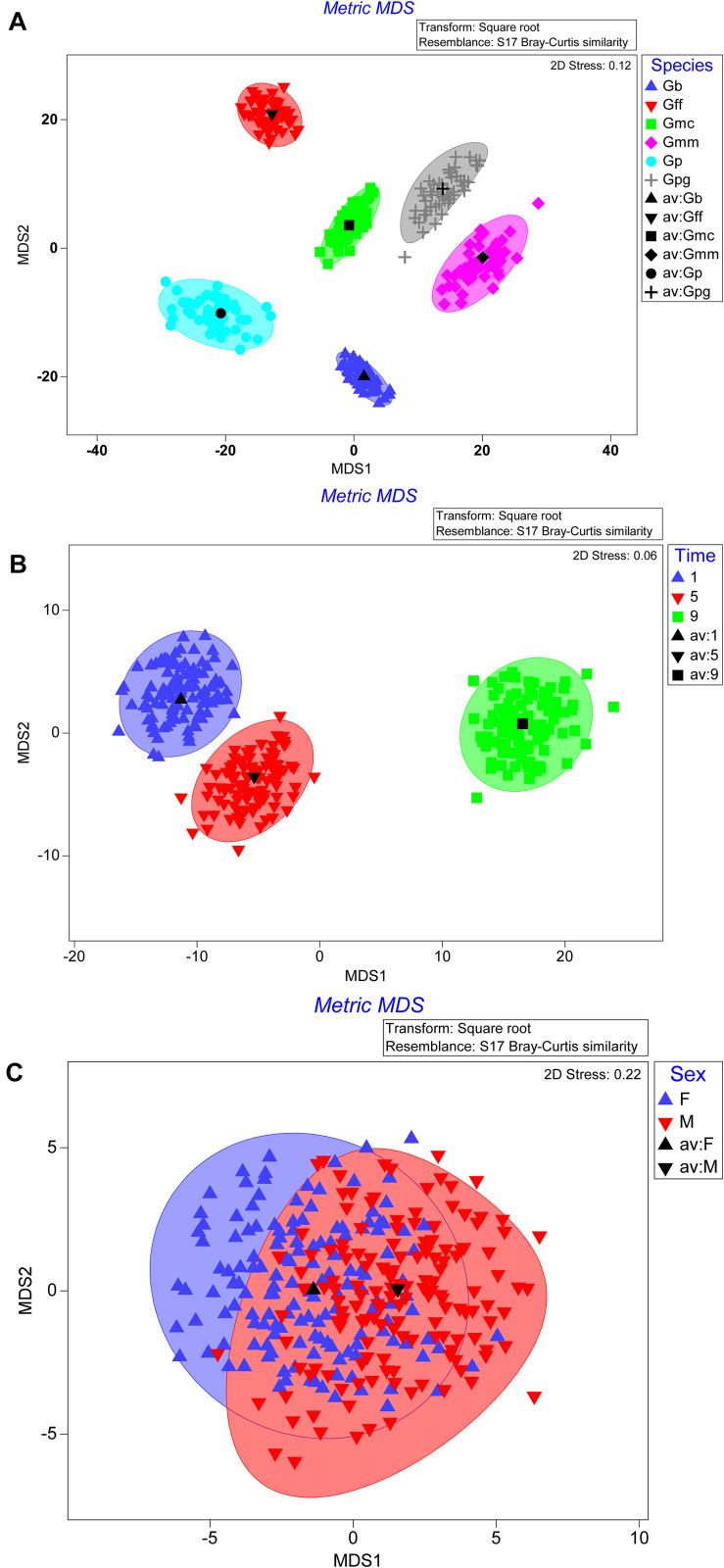
Metric multidimensional scaling (MMDS) of GpSGHV, *Wigglesworthia, Sodalis* and *Wolbachia* relative denisty with originations from tsetse species **(A)**, time post GpSGHV injection **(B)**, or sex **(C)**.

The bootstrap averages analysis of the metric multidimensional scaling (MMDS) produced clusters based on the species, the time post GpSGHV injection and the sex of the tested flies (Figure 4). Permanova analysis indicated that the clusters observed between tsetse species or the times post injection were statistically significant (*P* = 0.001); however, the clusters formed based on the sex level were not statistically significant (*P* = 0.735). The Permanova analysis also showed that there was a significant interaction between species and time post injection (*P* = 0.001) and sex (*P* = 0.002; Table 1).

**TABLE 1:** PERMANOVA results table for tsetse species, time post GpSGHV injection and sex and their combinations.

Source	*Df*	SS	MS	Pseudo-F	*P* (perm)	Unique perms
Species	5	1.05E+05	20,905	47.16	**0.001**	999
Time post injection	2	46,283	23,141	52.206	**0.001**	999
Sex	1	175.58	175.58	0.3961	0.735	999
Species × time post injection	10	49,580	4,958	11.185	**0.001**	996
species × sex	5	5,705	1,141	2.5741	**0.002**	998
Time post injection × sex	2	473.7	236.85	0.53433	0.775	999
Species × time post injection × sex	10	8,456.3	845.63	1.9077	**0.005**	999
Res	216	95,746	443.27			
Total	251	3.04E+05				

*Within the table, statistically significant differences (P < 0.05) can be seen in bold values in all three factors separately and in the combination of sex, species and time post injection. Perm(s) = permutation(s).*

## Discussion

In an attempt to improve our knowledge of GpSGHV infection, we investigated in the present study the relationship between GpSGHV infection and the three main bacterial symbionts of tsetse under laboratory rearing conditions. The demonstration of the virus infection and its density was previously investigated (Demirbas-Uzel et al., 2018b) and the impact of the virus infection on tsetse symbionts and the interaction between GpSGHV and symbionts was investigated in this study. The main findings can be summarized as follows: (a) the presence of the GpSGHV in artificially infected laboratory populations affects the densities of the three bacterial symbionts in a species-dependent manner while, in general, sex does not seem to play an important role; (b) with the exception of *G. fuscipes* where the high density of the GpSGHV was found in the presence of high density of *Sodalis*, there seems to be a negative correlation between GpSGHV and tsetse symbionts in all taxa; and (c) it seems that *G. pallidipes* have the lowest *Wigglesworthia* density compared to the other taxa. These findings suggest some possible explanations for the observed host range and variation in pathogenicity of the virus.

*Wigglesworthia* is a key factor for tsetse productivity and was found in all tested flies in the colonized species but with significant variation in the density. Similar to our results, *Wigglesworthia* has been detected in all field-collected samples that have been analyzed to date (Wang et al., 2013). Further, *Wig*Ds were found to be higher than the densities of *Sodalis* throughout all developmental stages in *G. p. gambiensis* (Soumana et al., 2013), which agrees with our results. Interestingly, in *G. f. fuscipes* and *G. g. gambiensis* infected with GpSGHV, *Wig*D was significantly reduced over time which might explain the result reported by Demirbas-Uzel et al. (2018c), which indicated a significant reduction in the flies performance in GpSGHV infected flies as compared with the control. In the same direction, the lowest *Wig*D was observed in *G. pallidipes*, a tsetse species which suffers most from the GpSGHV infection. These results support previous reports demonstrating the importance of *Wigglesworthia* for tsetse productivity and performance either by nutrition supplement or its immunological function in tsetse (Wang et al., 2009; Weiss et al., 2011). This might suggest that having low *Wig*D in *G. pallidipes* might compromise the flies’ immune system making it more vulnerable for GpSGHV infection and the development of SGH symptoms. An alternative explanation could be that the presence of GpSGHV reduces available nutritional resources required for *Wigglesworthia*’s multiplication leading to lower density. Our results in *G. f. fuscipes* also support the hypothesis that GpSGHV infection is associated with a significant reduction in *Wigglesworthia* density, which leads to a significant reduction in the flies’ productivity and performance (Demirbas-Uzel et al., 2018c), although SGH symptoms, similar to the ones observed in *G. pallidipes*, are not developed most likely due to low GpSGHV density (Abd-Alla et al., 2009a). The same hypothesis may be valid for *G. p. gambiensis* too; however, its validation will require the monitoring of *Wig*D in *G. f. fuscipes* and *G. p. gambiensis* for a longer period (beyond 9 dpi).

The increase in *Sodalis* densities over time in the flies injected with GpSGHV in *G. f. fuscipes* (significant increase) and *G. brevipalpis* (not significant) are in agreement with previous data reported by Demirbas-Uzel et al. (2018a), where *Sodalis* density increased in both males and females over time post emergence in a *G. m. morsitans* colony. However, the significant reduction in the *Sodalis* density in *G. p. gambiensis* and the non-significant decrease in *G. m. morsitans*, *G. m. centralis* and *G. pallidipes* contradicts the previously reported results of Demirbas-Uzel et al. (2018a). The increase of *Sodalis* density in *G. f. fuscipes* might be due to: (a) the reduction in *Wigglesworthia* density compromising the flies’ immune system and providing *Sodalis* with space and biological resources for multiplication and (b) the fact that the GpSGHV did not represent a competitive factor as its density remained below the density needed to develop SGH. However, the significant decrease in *Sod*D in *G. p. gambiensis* in GpSGHV infected flies was associated with a significant decrease in *Wig*D, which might indicate a different molecular mechanism governing the interaction between the endosymbiont, the GpSGHV and the host, most likely due to the fact that the GpSGHV infection in *G. p. gambiensis* is different from that in *G. f. fuscipes* (Demirbas-Uzel et al., 2018b). This also might be due to different strains of *Sodalis*, *Wigglesworthia* or GpSGHV circulating in the different tsetse species (Doudoumis et al., 2012; Kariithi et al., 2013).

Our results on the variations in the prevalence of *Sodalis* in the *Glossina* species is in accordance with previously reported data. For instance, prevalence of *Sodalis* was reported to be 93.7, 17.5, and 1.4% in wild-caught *G. brevipalpis*, *G. m. morsitans* and *G. pallidipes*, respectively (Wang et al., 2013; Dennis et al., 2014). Further, *Sodalis* prevalence varied widely (0–85%) across different wild-caught tsetse species (Wang et al., 2013). Although *Sodalis* lacks clearly defined functional roles in tsetse, it is interesting to note that in our study, the densities of this symbiont were significantly reduced in the virus-injected *G. p. gambiensis* compared to the controls. Another previous study showed that high *Sodalis* densities increased susceptibility of *G. morsitans* and *G. austeni* to pathogen (trypanosome) infection (Welburn et al., 1993; Dale and Welburn, 2001). In view of these previous findings, our results showing higher densities of *Sodalis* in virus-injected *G. f. fuscipes* could indicate an association between *Sodalis* and GpSGHV, however, the lack of increase in *Sod*D in *G. pallidipes* did not support some thoughts that *Sodalis* favors the establishment of GpSGHV infections in tsetse. The positive correlation between *Sodalis* and GpSGHV density in *G. f. fuscipes* only might be due to a different *Sodalis* strain circulating in *G. f. fuscipes* that coexists with GpSGHV at high density. In addition, the possibility that the GpSGHV injection induced a latent infection of a different strain of SGHV circulating in *G. f. fuscipes* should not be excluded.

*Wol*D varied significantly between tsetse taxa and the GpSGHV infection lead to a significant increase in *Wol*D in *G. f. fuscipes* and a significant reduction in *G. p. gambiensis*, whilst there was no significant alteration in other tsetse taxa. The molecular mechanism governing the dynamics of *Wolbachia* infection might be the same as for *Sodalis* (outlined above). These results confirm that tsetse flies, although they share many aspects of their biology, remain different in their interaction with bacterial symbionts and pathogens and the molecular mechanism for each species should be elucidated. Moreover, the interactions between symbionts, pathogens and their host are complex and remain to be uncovered.

Furthermore, *Wol*D was reported to differ not only between different tsetse host species but also between different populations within the same tsetse species (Alam et al., 2012; Doudoumis et al., 2012). Notably, in a previous investigation on the prevalence and co-infection dynamics among *Wolbachia*, GpSGHV and trypanosomes in wild-caught *G. f. fuscipes* (Alam et al., 2012), two findings were reported. First, in agreement with our findings, *Wolbachia* densities in wild *G. f. fuscipes* were at least 20-fold lower than in lab-bred *G. m. morsitans*. Since we found *Wolbachia* in all tested taxa, our results and the previous results imply that lab-bred flies may have higher *Wolbachia* densities than wild flies. The second finding was the negative correlation between the prevalence of GpSGHV infections and *Wolbachia* densities; our results appeared to agree with this since the high GpSGHV infections in *G. f. fuscipes* were accompanied by low *Wolbachia* titers over the 9 day period (except 0 dpi where the symbiont density were unexpectedly high).

The screening for coinfection with GpSGHV and each of the tsetse symbionts (*Wigglesworthia, Sodalis* and *Wolbachia)* in laboratory reared tsetse flies clearly indicated that the virus infection density in the tested tsetse species are independent. However, it seems that the virus infection might be controlled by tsetse symbionts regardless of tsetse taxa. Although coinfection can exist with low densities of both tsetse symbionts and GpSGHV, this does not exclude an antagonistic effect at higher density. Our data clearly show the absence of GpSGHV infection with high relative density in the presence of high density *Wigglesworthia* and *Wolbachia* infection. Moreover, a trend was also observed in *Sodalis* with the exception of the case of *G. f. fuscipes* where a high density of both GpSGHV and *Sodalis* was found. This might demonstrate the negative impact of *Wolbachia* on GpSGHV infection, in agreement with previous reports suggesting that the negative impact of *Wolbachia* on insect viruses is density dependent (Lu et al., 2012; Parry et al., 2019). The negative effect of *Wolbachia* on different RNA viruses has been well documented in mosquitoes and *Drosophila* (Hedges et al., 2008; Teixeira et al., 2008; Johnson, 2015) and this may be achieved by: (a) reducing virus replication and limiting virus transmission (Mousson et al., 2012); (b) supressing the expression of the DNA methyltransferase gene using a host microRNA to regulate its transcripts (Zhang et al., 2013); (c) activating antimicrobial peptides—defensins and cecropins (Pan et al., 2012) or (d) mediating antiviral activity, autophagy and iron metabolism and cholesterol and competition for the host cell (Rainey et al., 2014). In addition, there are several reports indicating that *Wolbachia* might enhance virus infection for both RNA and DNA viruses (Graham et al., 2012; Dodson et al., 2014). The above-mentioned mechanism might explain the possible antagonistic effect between *Wolbachia* and GpSGHV infection, however, this does not explain the observed antagonistic effect between *Wigglesworthis* and *Sodalis* (in most tsetse taxa) and GpSGHV infection. One possible mechanism is the competition for nutritional resources. This mechanism might also explain the observed dominance of one bacterium over other tsetse symbionts.

Taken together, available data show that the insect-virus-symbiont interactions are very delicate and complex, which in most cases only leave room for speculations on the mechanisms that are involved. In addition, the molecular dialogue between the tsetse host, bacterial symbionts, pathogens and parasites seems to be species specific and should not be a subject for broad generalization. In this context, the competition for nutritional resources between the symbionts and GpSGHV, as well as the potential presence and role of multiple strains of symbionts and GpSGHV circulating in different tsetse species, need to be considered. The coinfection of *Wigglesworthia, Sodalis*, *Wolbachia* and GpSGHV in laboratory reared flies seems to indicate an antagonistic effect at higher density, but in the light of the complicated situation observed in laboratory flies after GpSGHV infection, the impact of other elements, i.e., other virus infection or symbionts such as *Spiroplasma* etc., also needs to be investigated. In addition, the interactions of tsetse symbionts and GpSGHV in wild collected samples need to be analyzed. The lack of comprehensive and integrated considerations of tsetse physiology and behavior after GpSGHV infection has greatly limited our understanding of what appears to be evolutionarily significant host-virus-symbiont interactions and their underlying mechanisms. Further investigations are required to explore this topic.

## Conclusion

In summary, the results of the current study show that injecting the GpSGHV into tsetse flies leads to variable responses in *Wigglesworthia, Sodalis* and *Wolbachia* dependent on the taxon. This indicates that the interaction and molecular dialogue between the host and its symbionts, parasites and pathogens is a complicated and species-specific process. At high infection density *Wigglesworthia*, *Sodalis* (in most tsetse taxa except *G. f. fuscipes*) and *Wolbachia* was associated with relatively low GpSGHV density. The low density of *Wigglesworthia* in *G. pallidipes* might explain the severe impact of GpSGHV infection on this species. As tsetse symbionts are more prevalent in colonized tsetse flies and their density may increase over time, this might help tsetse species avoid GpSGHV infection and thereby ensure sustainable production of sterile males for the implementation of SIT programs.

## Data Availability Statement

The datasets presented in this study can be found in online repositories. The names of the repository/repositories and accession number(s) can be found below: https://dataverse.harvard.edu/dataset.xhtml?persistentId=doi:10.7910/DVN/X15PQF.

## Author Contributions

GD-U performed the experiments, analyzed the data, and drafted the manuscript. AA and VD performed the experiments and critically revised the manuscript. AP performed statistical analysis and critically revised the manuscript. GT critically revised the manuscript. KB conceived the study, designed the experiments, interpreted the data, contributed to the drafting, and critically revised the manuscript. AA-A conceived the study, designed the experiments, interpreted the data, and draft the manuscript. All authors approved the final version of the manuscript and agreed to be accountable for all aspects of the work in ensuring that questions related to the accuracy or integrity of any part of the work are appropriately investigated and resolved.

## Conflict of Interest

The authors declare that the research was conducted in the absence of any commercial or financial relationships that could be construed as a potential conflict of interest.

## Supplementary Material

Supplementary Figure 1 Relative density of GpSGHV in six taxa of tsetse flies over days post injection. Transformed GpSGHV density for six taxa of tsetse flies injected with GpSGHV normalized against control flies. Total DNA was extracted from six females and two males at 1, 5, and 9 days post injection and used as template for qPCR. Data were normalized to the housekeeping β*-tubulin* gene, against time zero and against the control injected flies at each time point and transformed based on the Box-Cox lambda value. The linear regression line was calculated using the lm method in R.

Supplementary Figure 2 Heatmap indicating the relative density of tsetse endosymbionts *Wigglesworthia* **(A)**, *Sodalis* **(B),** and *Wolbachia* **(C)** between males and females in adult tsetse flies of six species injected with GpSGHV.
