# Supplementary figures and images for: Interactions Between Tsetse Endosymbionts and Glossina pallidipes Salivary Gland Hypertrophy Virus in Glossina Hosts
Source: Front Microbiol. 2021 May 28;12:653880. doi: 10.3389/fmicb.2021.653880 (PMC8194091; doi:10.3389/fmicb.2021.653880)

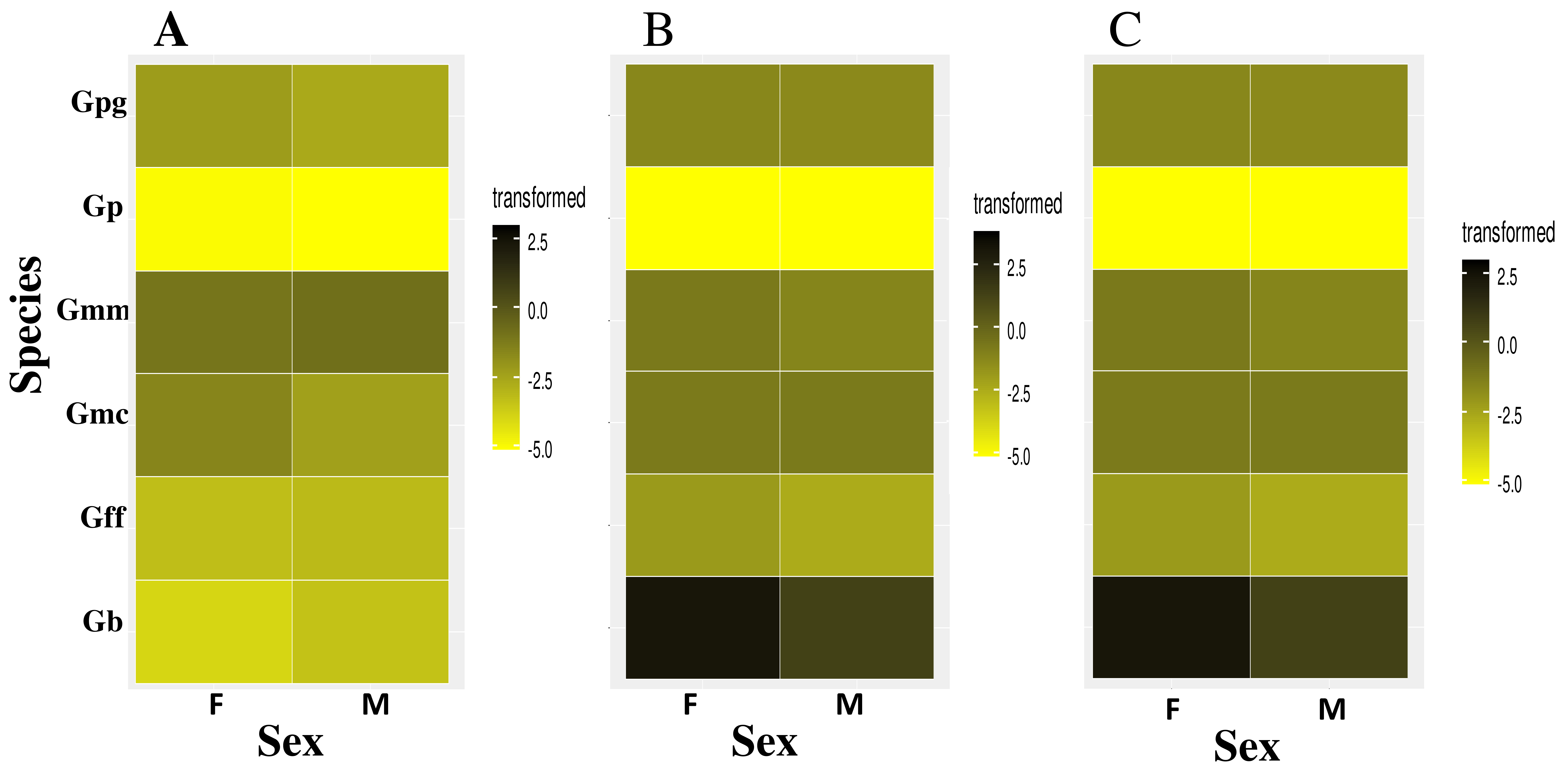

Supplement: Supplementary Figure 2 — Heatmap indicating the relative density of tsetse endosymbionts Wigglesworthia (A), Sodalis (B), and Wolbachia (C) between males and females in adult tsetse flies of six species injected with GpSGHV. [file Image_2.TIF]
