# Supplementary material for: Interactions Between Tsetse Endosymbionts and Glossina pallidipes Salivary Gland Hypertrophy Virus in Glossina Hosts
Source: Front Microbiol. 2021 May 28;12:653880. doi: 10.3389/fmicb.2021.653880 (PMC8194091; doi:10.3389/fmicb.2021.653880)
Supplement: Supplementary file 3 [file Data_Sheet_1.docx]

**Supplementary Tables**

**Supplementary Table 1.** List of Primers used for quantitative PCR (qPCR) analyses of microbiome in *Glossina* species

| **Target Gene** | **Primer Name** | **Primer Sequence**  **(Listed 5- to -3)** | **Annealing Temperature (^°^C)** | **Amplicon Size (bp)** | **References** |
| --- | --- | --- | --- | --- | --- |
| fliC (flagellin) (Sodalis) | sodqPCR-FliCF | GAA GCC ACC GAT CCT GTA AC | 55 | 508 | (Weiss et al., 2012)] |
|  | sodqPCR-FliCR | CAT CTT TGC CCG TAG AAA TCA C |  |  |  |
| Codhoc (Wigglesworthia) | WiggqPCRcodhocF2 | GACTTGTACGTGATATTTCCAAGC | 60 | 645 | (Rose et al., 1998) |
|  | WiggqPCRcodhocR2 | GACATCAAATCGCGTTACTGG |  |  |  |
| Wolbachia 16S rRNA  (Wolbachia) | Wsp fwd | YATACCTATTCGAAGGGATAGAGCTTCGAGTGAA ACCAATTC | 60 | 282 | (Doudoumis et al., 2012; Brelsfoard et al., 2014) |
|  | Woltse- cyt R | GGATTAGCTTAGCCTCGC |  |  |  |
| β-tubulin  (Tsetse Fly) | Tsetse-tubulinF | GAT GGT CAA GTG CGA TCC T | 55 | 355 | (Caljon et al., 2009) |
|  | Tsetse-tubulinR | TGA GAA CTC GCC TTC TTC C |  |  |  |
| thiC (thiamine  biosynthesis)  (Wigglesworthia) | WiggqPCRthiCF | GACATCAAATCGCGTTACTGG | 60 | 645 | (Boucias et al., 2013) |
|  | WiggqPCRthiCR | GACTTGTACGTGATATTTCCAAGC' |  |  |  |
| odv-e66 (GpSGHV ORF5) | qPCRFwda | CAAATGATCCGTCGTGGTAGAA | 60 | 51 | (Abd-Alla et al., 2009, 2011) |
|  | qPCRFwda | CAAATGATCCGTCGTGGTAGAA |  |  |  |
| wsp (16S ribosomal RNA) | Wsp fwd | YATACCTATTCGAAGGGATAG | 55 | *438* | (Werren and Windsor, 2000; Doudoumis et al., 2012) |
|  | Wsp rev | AGCTTCGAGTGAAACCAATTC |  |  |  |
| odv-e66 (GpSGHV ORF5) | GpSGHV2Fwd | CTTGTCAGCGCCACGTACAT | 55 | 401 | (Abd-Alla et al., 2007) |
|  | GpSGHV2Rev | GCATTCACAGCATCCCAATTTT |  |  |  |

Reference list

Abd-Alla, A., Bossin, H., Cousserans, F., Parker, A., Bergoin, M., and Robinson, A. (2007). Development of a non-destructive PCR method for detection of the salivary gland hypertrophy virus (SGHV) in tsetse flies. *J. Virol. Methods* 139, 143–149. Available at: DOI: 10.1016/j.jviromet.2006.09.018.

Abd-Alla, A. M. M., Cousserans, F., Parker, A., Bergoin, M., Chiraz, J., and Robinson, A. (2009). Quantitative PCR analysis of the salivary gland hypertrophy virus (GpSGHV) in a laboratory colony of *Glossina pallidipes*. *Virus Res.* 139, 48–53. Available at: http://dx.doi.org/10.1016/j.virusres.2008.10.006.

Abd-Alla, A. M. M., Salem, T. Z., Parker, A. G., Wang, Y., Jehle, J. A., Vreysen, M. J. B., et al. (2011). Universal primers for rapid detection of hytrosaviruses. *J Virol Methods* 171, 280–283. Available at: http://dx.doi.org/10.1016/j.jviromet.2010.09.025.

Boucias, D. G., Kariithi, H. M., Bourtzis, K., Schneider, D. I., Kelley, K., Miller, W. J., et al. (2013). Transgenerational transmission of the *Glossina pallidipes* hytrosavirus depends on the presence of a functional symbiome. *PLoS One* 8, e61150-. Available at: http://dx.doi.org/10.1371/journal.pone.0061150.

Brelsfoard, C., Tsiamis, G., Falchetto, M., Gomulski, L. M., Telleria, E., Alam, U., et al. (2014). Presence of extensive *Wolbachia* symbiont insertions discovered in the genome of its host *Glossina morsitans morsitans*. *PLoS Negl. Trop. Dis.* 8, e2728-. Available at: DOI:10.1371/journal.pntd.0002728.

Caljon, G., Broos, K., Goeyse, I. D., De Ridder, K., Sternberg, J. M., Coosemans, M., et al. (2009). Identification of a functional Antigen5-related allergen in the saliva of a blood feeding insect, the tsetse fly. *Insect Biochem. Mol. Biol.* 39, 332–341.

Doudoumis, V., Tsiamis, G., Wamwiri, F., Brelsfoard, C., Alam, U., Aksoy, E., et al. (2012). Detection and characterization of *Wolbachia* infections in laboratory and natural populations of different species of tsetse flies (genus *Glossina)*. *BMC Micobiology* 12, S3-. Available at: doi:10.1186/1471-2180-12-S1-S3.

Rose, T. M., Schultz, E. R., Henikoff, J. G., Pietrokovski, S., McCallum, C. M., and Henikoff, S. (1998). Consensus-degenerate hybrid oligonucleotide primers for amplification of distantly related sequences. *Nucleic Acids Res* 26, 1628–1635.

Weiss, B. L., Maltz, M., and Aksoy, S. (2012). Obligate symbionts activate immune system development in the tsetse fly. *J Immunol* 188, 3395–3403. Available at: doi: 10.4049/?jimmunol.1103691.

Werren, J. H., and Windsor, D. M. (2000). *Wolbachia* infection frequencies in insects: evidence of a global equilibrium? *Proc Biol Sci* 267, 1277–1285. Available at: doi: 10.1098/rspb.2000.1139.

**Supplementary Table 2**. ANOVA of the transformed *Wigglesworthia*, *Sodalis* and *Wolbachia* density relative to PBS

| **Treatment** | **Interactions** | **F** | **df** | **P value** |
| --- | --- | --- | --- | --- |
| *Wigglesworthia* (for 6 taxa) | Sex | 0.023 | 1,206 | 0.881 |
|  | Time | 5.300 | 1,206 | 0.0223* |
|  | Taxon | 42.855 | 5,206 | < 2.2e-16 *** |
|  | Sex-Time | 2.747 | 1,206 | 0.0990 |
|  | Sex-Taxon | 7.014 | 5,206 | 4.48e-06 *** |
|  | Time-Taxon | 3.318 | 5,206 | 0.00665 ** |
|  | Sex-Time-Taxon | 1.671 | 5,206 | 0.143 |
| *Sodalis* (for 6 taxa) | Sex | 2.321 | 1,228 | 0.129 |
|  | Time | 0.876 | 1,228 | 0.350 |
|  | Taxon | 70.593 | 5,228 | < 2.2e-16 *** |
|  | Sex-Time | 5.819 | 1, 228 | 0.017* |
|  | Sex-Taxon | 4.417 | 5,228 | 0.000738 *** |
|  | Time-Taxon | 8.778 | 5,228 | 1.24e-07 *** |
|  | Sex-Time-Taxon | 6.899 | 5,228 | 5.10e-06 *** |
| *Wolbachia*  (for 6 taxa) | Sex | 1.399 | 1,189 | 0.238 |
|  | Time | 0.067 | 1,189 | 0.796 |
|  | Taxon | 20.868 | 5,189 | < 2e-16 *** |
|  | Sex-Time | 0.007 | 5,189 | 0.934 |
|  | Sex-Taxon | 1.378 | 5,189 | 0.234 |
|  | Time-Taxon | 1.891 | 5,189 | 0.098 |
|  | Sex-Time-Taxon | 1.165 | 5,189 | 0.328 |
|  | Sex | 1.0497 | 1,210 | 0.3068 |
|  | Time | 220.089 | 1,210 | <2e-16 *** |
| GpSGHV | Taxon | 31.7112 | 5,210 | <2e-16 *** |
|  | Sex-Time | 0.0318 | 1,210 | 0.8586 |
|  | Sex-Taxon | 1.0558 | 5,210 | 0.3860 |
|  | Time-Taxon | 27.025 | 5,210 | <2e-16 *** |
|  | Sex-Time-Taxon | 0.7744 | 5,210 | 0.5692 |

Signif. codes: ‘***’ *P <* 0.001; ‘**’ *P <* 0.01; ‘*’ *P <* 0.05

**Supplementary Table 3.** ANOVA Statistics between Female and Male for *Wigglesworthia, Sodalis* and *Wolbachia* density on laboratory tsetse taxa

| **Bacterium** | **Taxon** | **F** | **df** | **P value** |
| --- | --- | --- | --- | --- |
| *Wigglesworthia* | *G. brevipalpis* | 9.537 | 1,52 | 0.00323 ** |
|  | *G. f. fuscipes* | 2.039 | 1,34 | 0.162 |
|  | *G. m. centralis* | 12.407 | 1,34 | 0.00124 ** |
|  | *G. m. morsitans* | 3.688 | 1,34 | 0.0632 |
|  | *G. pallidipes* | 2.071 | 1,30 | 0.161 |
|  | *G. p. gambiensis* | 2.784 | 1,34 | 0.104 |
| *Sodalis* | *G. brevipalpis* | 2.420 | 1,52 | 0.126 |
|  | *G. f. fuscipes* | 0.294 | 1,52 | 0.590 |
|  | *G. m. centralis* | 21.799 | 1,34 | 4.60e-05 *** |
|  | *G. m. morsitans* | 0.597 | 1,34 | 0.445 |
|  | *G. pallidipes* | 0.234 | 1,34 | 0.631 |
|  | *G. p. gambiensis* | 22.147 | 1,34 | 4.12e-05 *** |
| *Wolbachia* | *G. brevipalpis* | 5.977 | 1,52 | 0.0179 * |
|  | *G. f. fuscipes* | 0.416 | 1,34 | 0.523 |
|  | *G. m. centralis* | 0.236 | 1,16 | 0.634 |
|  | *G. m. morsitans* | 1.478 | 1,31 | 0.233 |
|  | *G. pallidipes* | 0.212 | 1,34 | 0.649 |
|  | *G. p. gambiensis* | 3.910 | 1,34 | 0.0562 |
|  | *G. brevipalpis* | 2.2624 | 1,50 | 0.1388 |
|  | *G. f. fuscipes* | 2.5393 | 1,32 | 0.1209 |
| GpSGHV | *G. m. centralis* | 0.4411 | 1,32 | 0.5113 |
|  | *G. m. morsitans* | 0.0457 | 1,32 | 0.8321 |
|  | *G. pallidipes* | 0.0115 | 1,32 | 0.9153 |
|  | *G. p. gambiensis* | 0.0441 | 1,32 | 0.8349 |

Signif. codes: ‘***’ *P <* 0.001; ‘**’ *P <* 0.01; ‘*’ *P <* 0.05

**Supplementary Table 4**. Statistics values for the regression analysis for the transformed density of *Wigglesworthia*, *Sodalis* and *Wolbachia* after normalizing the data of each time point against the data of zero-time and normalizing the data of each point of the virus injected samples against the same time point of the PBS infected samples.

|  |  | **Regression (Female and male)** | | | | | | |
| --- | --- | --- | --- | --- | --- | --- | --- | --- |
| **Bacterium** | **Tsetse Taxon** | Coefficient | t value | *P* value | Intercept | Intercept t value | Intercept  *P* value | R^2^ |
| *Wigglesworthia* | *G. brevipalpis* | 0.031 | 0.418 | 0.677 | -1.204 | -2.751 | 0.008 ** | -0.015 |
|  | *G. f. fuscipes* | -0.165 | -3.447 | 0.001 ** | -0.910 | -3.181 | 0.003 ** | 0.237 |
|  | *G. m. centralis* | 0.014 | 0.271 | 0.788 | -0.698 | -2.240 | 0.032 * | -0.027 |
|  | *G. m. morsitans* | -0.063 | -1.411 | 0.167 | 0.354 | 1.325 | 0.194 | 0.028 |
|  | *G. pallidipes* | 0.237 | 0.999 | 0.326 | -3.975 | -10.528 | 1.36e-11 *** | -7.39e-05 |
|  | *G. p. gambiensis* | -0.170 | -3.368 | 0.002 ** | 0.060 | 0.199 | 0.843 | 0.228 |
| *Sodalis* | *G. brevipalpis* | 0.034 | 1.724 | 0.0906 | -0.293 | -2.497 | 0.016 * | 0.036 |
|  | *G. f. fuscipes* | 0.071 | 4.088 | < 0.001 *** | 0.338 | 3.257 | 0.002 ** | 0.229 |
|  | *G. m. centralis* | -0.014 | -1.182 | 0.245 | 0.298 | 4.296 | < 0.001 *** | 0.011 |
|  | *G. m. morsitans* | -0.040 | -1.644 | 0.110 | -0.277 | -1.898 | 0.066 | 0.046 |
|  | *G. pallidipes* | -0.010 | -0.814 | 0.422 | -0.289 | -4.148 | < 0.001 *** | -0.010 |
|  | *G. p. gambiensis* | -0.051 | -3.624 | < 0.001 *** | 0.114 | 1.376 | 0.178 | 0.258 |
| *Wolbachia* | *G. brevipalpis* | -0.036 | -0.036 | 0.629 | 0.982 | 2.263 | 0.028 * | -0.015 |
|  | *G. f. fuscipes* | 0.224 | 3.079 | 0.004 ** | -3.123 | -7.187 | 2.59e-08 *** | 0.195 |
|  | *G. m. centralis* | -0.026 | -1.478 | 0.159 | -0.394 | -3.736 | 0.002 ** | 0.065 |
|  | *G. m. morsitans* | -0.047 | -0.661 | 0.513 | -0.219 | -0.497 | 0.623 | -0.018 |
|  | *G. pallidipes* | 0.066 | 0.422 | 0.676 | -2.987 | -3.209 | 0.003 ** | -0.024 |
|  | *G. p. gambiensis* | -0.125 | -2.767 | 0.009 *** | -0.102 | -0.378 | 0.708 | 0.160 |
|  | *G. brevipalpis* | -0.01516 | -0.0135 | 0.429 | -0.013 | -0.121 | 0.904 | -0.0069 |
|  | *G. f. fuscipes* | 0.09141 | 0.949 | 0.349 | 0.72250 | 1.256 | 0.218 | -0.0028 |
| GpSGHV | *G. m. centralis* | 0.56227 | 10.786 | < 0.001 *** | -1.89884 | -6.099 | 6.41e-07 *** | 0.7672 |
|  | *G. m. morsitans* | 0.79789 | 13.346 | 4.49e-15 *** | -1.87347 | -5.247 | 8.21e-06 *** | 0.835 |
|  | *G. pallidipes* | 0.40981 | 4.911 | 2.24e-05 *** | -2.48034 | -4.977 | -4.977 | -4.977 |
|  | *G. p. gambiensis* | 0.8349 | 10.376 | 4.5e-12 *** | -0.35703 | -1.146 | 0.26 | 0.7529 |

Signif. codes: ‘***’ *P <* 0.001; ‘**’ *P <* 0.01; ‘*’ *P <* 0.0%
